# Suppression of (0001) plane emission in GaInN/GaN multi-quantum nanowires for efficient micro-LEDs

**DOI:** 10.1515/nanoph-2022-0388

**Published:** 2022-09-28

**Authors:** Sae Katsuro, Weifang Lu, Kazuma Ito, Nanami Nakayama, Shiori Yamamura, Yukimi Jinno, Soma Inaba, Ayaka Shima, Naoki Sone, Dong-Pyo Han, Kai Huang, Motoaki Iwaya, Tetsuya Takeuchi, Satoshi Kamiyama

**Affiliations:** Department of Materials Science and Engineering, Meijo University, 1-501 Shiogamaguchi, Tenpaku-ku, Nagoya, 468-8502, Japan; Fujian Key Laboratory of Semiconductor Materials and Applications, CI Center for OSED, Department of Physics, Xiamen University, Xiamen 361005, China; Future Display Institute in Xiamen, Xiamen 361005, China

**Keywords:** (0001)-plane emission, dry etching, GaInN/GaN, micro-LED, multi-quantum-shell, nanowire

## Abstract

GaInN/GaN multi-quantum-shell (MQS) nanowires (NWs) are gaining increasing attention as promising materials for developing highly efficient long-wavelength micro-light emitting diodes (LEDs). To improve the emission properties in GaInN/GaN MQS NWs, it is necessary to suppress the emission from the (0001) *c*-plane MQS at the apex region, which featured with low crystalline quality. In this study, we investigated the enhancement of optical properties and the realization of micro-LEDs by confirming the effect of the (0001) plane region. A 7.9-fold enhancement of the electroluminescence (EL) intensity was demonstrated by removal the (0001) plane region via inductively coupled plasma (ICP) dry etching, owing to the promoted current injection into the (1–101) semi-polar and (10–10) non-polar sidewall area. To investigate the effect of the emission area on the samples with and without truncated (0001) plane region, devices with three different mesa areas (50 × 50, 100 × 100, and 100 × 200 μm^2^) were fabricated. An increased EL intensity with the reduced mesa areas was observed in the samples without dry etching of the (0001)-plane area, because more current can be injected into the sidewall region with higher crystalline quality and luminous efficiency than the (0001)-plane MQS. Under the same injection current density, the truncated samples’ light output was increased for more than ten times as compared to the samples without (0001)-plane etching. Therefore, it confirms the possibility of realizing highly efficient GaInN/GaN MQS NWs LEDs by eliminating the (0001) plane MQS region. A precise etching and surface passivation of the apex region is expected to further reduce the reverse leakage current and improve the performance in NW-LEDs.

## 1 Introduction

Researchers are working to substantially reduce the chip size of gallium nitride (GaN)-based devices to broaden the application range of these devices under the front-end economic driving. Among the cutting-edge technologies, micro-light emitting diodes (micro-LEDs) are considered as the next generation of display systems, as they can outperform organic LEDs displays in terms of durability, excellent stability, higher reliability, and outstanding efficiency [1–4]. Regarding the chip design for high efficiency micro-LEDs, thermal saturation and efficiency droop caused by auger recombination, the reduction of the maximum value of external quantum efficiency and wall-plug efficiency, and the surface recombination at the edge of the active layer region caused by the chip size reduction are some of the key obstacles [5–7]. Moreover, to realize GaInN/GaN based long wavelength micro-LEDs, it is inevitable to solve the commonly existing “green gap” problem at the emission wavelength range between 535 and 570 nm [8–10]. LED structures grown on a polar (0001) *c*-plane substrate involves a strain-induced piezoelectric field due to the lattice mismatch between GaInN and GaN, which increases with an increase in InN fraction [11, 12]. Furthermore, the spontaneous polarization of the (0001) plane likewise reduces the recombination efficiency of the electrons and holes, resulting in a decrease in emission efficiency [13]. Alternatively, GaN/GaInN nanowires (NWs), which are hexagonal prismatic microcrystals of nitride semiconductors, are promising for the realization of highly efficient long-wavelength LEDs [14–23]. Three-dimensional structures that possess (10–10) *m*-plane (non-polar plane) and (1–101) plane (semi-polar plane) on the sidewalls with the increased emitting area because the coaxially GaN/GaInN based multiple quantum shell (MQS) NWs are featured with low-dislocation density [14, 24], and enable control of the emission wavelength [25–29]. Furthermore, the coaxial MQS structure in NW-LEDs enable minimizing the exposed area of active region after mesa etching, which can effectively prevent the high surface recombination rate near the edge of the mesa due to the surface damage, dangling bonds, and other surface defects [30].

Nevertheless, due to the intrinsic spatial distribution of InN fraction along with the hexagonal prismatic NW structures, it is difficult to achieve conformal deposition of thin indium thin oxide (ITO) on the NWs for current spreading [31] and maintain high emission stability when fabricating NW-LED devices [32, 33]. When an optimal growth condition with an enhanced growth rate in the lateral direction was applied, the p-GaN shell on NWs appeared like a trigonal pyramid, improving the conformal ITO deposition and making NWs less susceptible to breakage [34]. For MQS structures, crystal growth is simultaneously performed under identical conditions. However, due to spatial differences in incorporation and diffusion distances, the emission wavelengths shorten from the (0001) plane at the apex region to (1–101) and (10–10) planes near the sidewall [25]. Even though the emission from the (1–101) plane is dominant under low injection current, NW-LEDs using semipolar (1–101) planes luminescence area are also promising for realizing high-efficiency long-wavelength emission, as the (1–101) plane at apex region manifests a higher In incorporation than the bottom area [32]. The (0001) plane MQS has a higher growth rate than the other regions in the apex region of NWs, reducing desorption of In during growth. Moreover, the MQS thickness on (0001) plane region exceeds the critical thickness of the two-dimensional growth mode, which can induce several misfit dislocations [35, 36]. Meanwhile, thermally decomposed parts with high In content and unstable three-dimensional surfaces are easily formed, and thus, In-rich clusters along with serpentine-shaped morphology were commonly observed there [34]. Therefore, the emission from the (0001) plane region of the NWs is influenced by the quantum confined stark effect (QCSE) related blueshift, nonradiative recombination, and deep level defects, leading to a decrease in luminous efficiency [37, 38]. Furthermore, there is a concern about current leakage caused by the defects in p-GaN near the (0001) plane region [32, 39]. To some extent, it has been verified that the improvement of crystalline quality of the p-GaN shell, especially at (0001) plane region, can mitigate the localization of injection current at the apex region [32, 34]. Nevertheless, the emission components from (0001) plane MQS region inevitably exist in NW-LEDs, which is not preferred for high stability micro-LEDs. Thus far, research results regarding the suppression of the (0001) plane emission in NW-LEDs have not been reported yet.

In this study, to improve the emission properties of GaInN/GaN MQS NW-LEDs, we reported on an effective suppression method of the current injection into the apex of NWs by dry etching of the (0001) plane region. For comparison, the dependence of chip size on the emission properties of NW-LEDs with and without truncated (0001) plane region was also investigated. The morphologies of the as-grown NWs and variation during the device fabrication process were examined using scanning transmission electron microscope (SEM) measurements. The electronic and emission properties were analyzed based on the results of current–voltage–light output characteristics, electroluminescence (EL) spectra, and cathodoluminescence (CL) spectra of the NW-LEDs. It showed a high possibility of precisely removing the (0001) plane MQS region of NWs, and thereby suppressing the relevant emission component to realize high-efficiency NW-LEDs.

## 2 Experimental section

**Figure 1:**
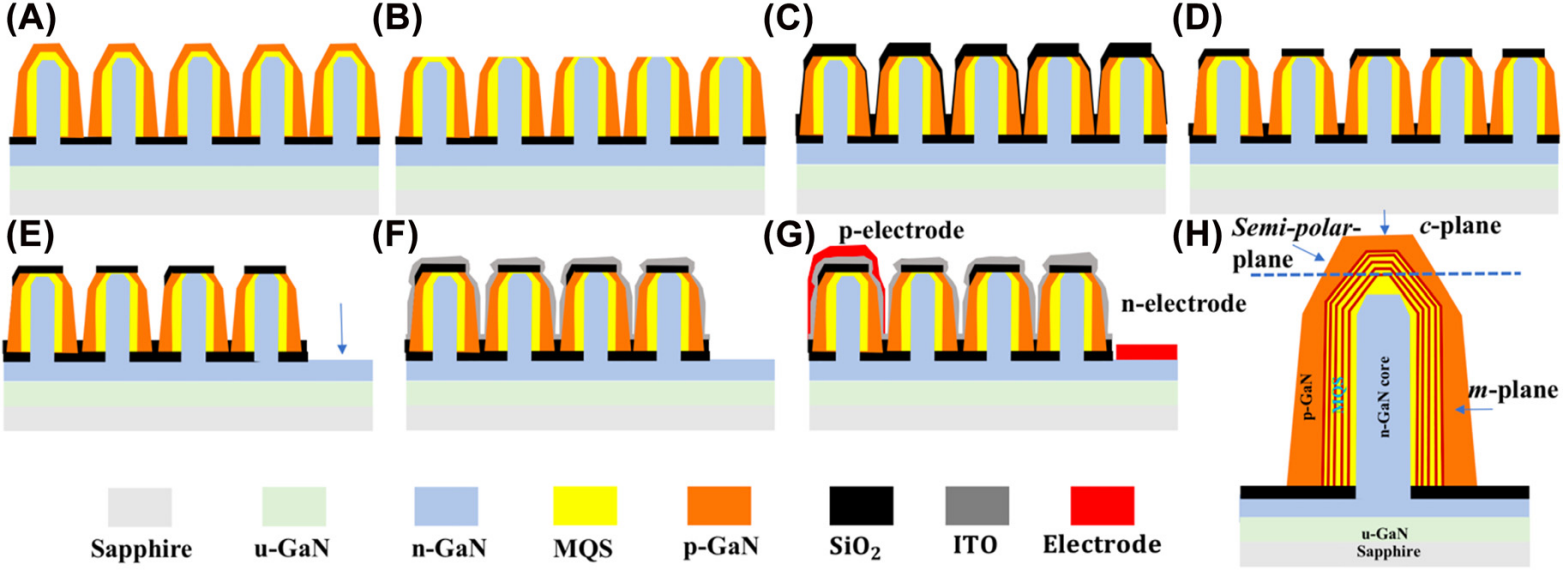
Schematic diagrams of the NW-LED fabrication process. (A) MOVPE-grown NW samples with activation annealing of p-GaN by RTA; (B) ICP dry etching to selectively remove the (0001) plane MQS at the apex region of the NWs; (C) deposition of SiO_2_ insulating film on NWs; (D) SiO_2_ deposited on (1–101) and (10–10) planes sidewall was wet etched by buffered hydrofluoric acid (BHF); (E) removal of NWs in n-electrode region and exposure of n-GaN layer by ICP dry etching; (F) sputtering of 60 nm-thick ITO layer on the NWs in mesa region, followed by a thermal annealing process at 600 °C for 4 min in an N_2_ atmosphere; (G) deposition of the Cr/Ni/Au films with a respective thickness of 10, 20, and 200 nm on the n- and p-electrodes; (H) cross-sectional view of NW structures with etching mark of the (0001) plane MQS at the apex region.

Metalorganic vapor epitaxy (MOVPE) with selective growth was used to fabricate the samples used for the NW-LED process in this study [40, 41]. First, a SiO_2_ mask layer was deposited on the n-GaN/sapphire substrate templates using a radiofrequency magnetron sputtering system (CFS-4EP, Shibaura Mechatronics Co, Yokohama City, Kanagawa, Japan). The deposition was conducted under the condition of RF power of 450 W and Ar atmosphere, aiming at 30 nm thickness. Using nano-imprint lithography (NIL) technology, a triangular lattice hole pattern with a diameter of 300 nm and a pitch of 1200 nm was formed. The hole pattern was exposed by inductively coupled plasma (ICP) etching (MV06-7001-0, ULVAC, Inc., Chigasaki City, Kanagawa Japan) under ambient CF_4_ gas. Subsequently, Si-doped n-GaN core NWs were grown at a growth temperature of 1135 °C for 70 s, followed by five pairs of GaInN/GaN MQS with AlGaN spacers [17]. An optimized growth sequence of conformal p-GaN shells was grown on the NWs, as reported in our previous work [34]. After the epitaxial growth of NW samples, two batches of LED devices were fabricated using the standard fabrication processes without (samples a, b, and c) and with removing the (0001) plane MQS region on the top of NWs by ICP dry etching (samples d, e, and f). The corresponding device sizes of the samples are shown in Table 1. Figure 1 schematically depicts the device fabrication process and the cross-sectional view of the NW structures. To activate the p-GaN shell on NWs [see Figure 1(A)], thermal annealing of p-GaN for all the samples was performed at 650 °C for 30 min under N_2_ and O_2_ atmosphere by rapid thermal processing (RTA) (MR094017-0, ULVACRIKO, INC., Tsuzuki City, Yokohama, Japan). Subsequently, the NW-LED samples a, b, and c underwent the standard fabrication process, including the exposure of the n-electrode area, ITO deposition on the mesa area, and metallic Cr/Ni/Au (with a thickness of 10, 20, and 200 nm, respectively) films evaporation for n/p-electrodes, as shown in Figure 1(E–G), respectively. Here, to selectively expose the n-electrode region, the NWs of the other region were protected by an electron beam (EB) evaporated (MA08-3065, ULVAC, Inc., Chigasaki City, Kanagawa, Japan) Ni layer with a thickness of 200 nm. For the case with apex etching, the (0001) plane MQS of the NWs in samples d, e, and f was removed beforehand by ICP dry etching with an etching rate ratio of 1:1 between the GaN and resist, as depicted in Figure 1(B). Subsequently, a 160 nm-thick SiO_2_ layer was deposited to insulate the exposed apex region of the NWs by setting the rotation speed of the radiofrequency magnetron sputtering system to 0, as shown in Figure 1(C). During sputtering, the plasma ejected microscopic particles of solid SiO_2_ moved toward the NW samples on the rotating susceptor, resulting in the deposition from different directions. Under the rotation speed of zero, the SiO_2_ material was vertically sputtered on the top of the NWs and mask area. A 100–130 nm-thick SiO_2_ is required to effectively insulate the exposed apex region, and here the 160 nm-thick layer was deposited in anticipation for subsequent removing of SiO_2_ on the sidewalls by wet etching. To dissolve the SiO_2_ deposited on the (1–101) and (10–10) plane sidewall, the samples d, e, and f were dipped in the dilute buffered hydrofluoric acid (BHF) (diluted 1: 100 in deionized water). The subsequent fabrication process for samples d, e, and f was identical to that of samples a, b, and c.

**Table 1:** NW-LED samples with/without dry etching of (0001) plane area and the corresponding device size.

Sample	a	b	c	d	e	f
With or without (0001) plane etching	Without	Without	Without	With	With	With
Mesa area (µm^2^)	50 × 50	100 × 100	200 × 100	50 × 50	100 × 100	200 × 100
p-electrode area (µm^2^)	1600	1600	5400	1600	1600	5400
Ratio of p-electrode to mesa area	0.64	0.16	0.27	0.64	0.16	0.27
Images of the devices	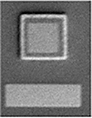	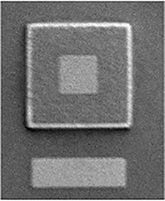	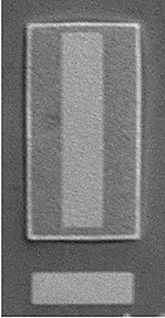	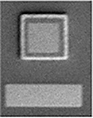	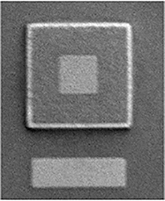	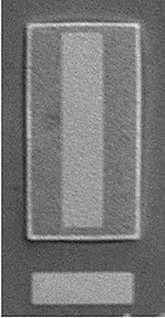

During the fabrication process, the structural morphologies of the NWs were inspected by an SEM system (SU70, Hitachi High-Technologies Co., Minato City, Tokyo, Japan) with an acceleration voltage of 3 V. The optical-electrical characterizations of the fabricated NW-LEDs were performed by EL (Ocean Optics Co., United States), CL (SEM-SU 50000, Hitachi Co., Minato City, Tokyo, Japan), and current–voltage–light output (*I*–*V*–*L*) (4156c, Agilent Technology, Santa Clara CA, USA) measurements. Regarding the EL measurement, the light emission signal of all the NWs in one chip was detected from the front side under a constant current. In contrast, the CL measurement was performed on the cross-section of a single NW with an EB irradiation, and the emission signal from each plane [(0001), (1–101), and (10–10) planes] of the NW was collected. Therefore, it is possible to assign the dominant emission planes of the NWs for the EL spectra by comparing them with the CL results. The intensity of EL and *I*–*V*–*L* results are comparable because the signal was acquired from the rear side of the NW-LEDs via fixed detectors. Furthermore, the all measurements were performed under the identical conditions.

## 3 Results and discussion

### 3.1 Inspection of NW structures during LED fabrication

**Figure 2:**
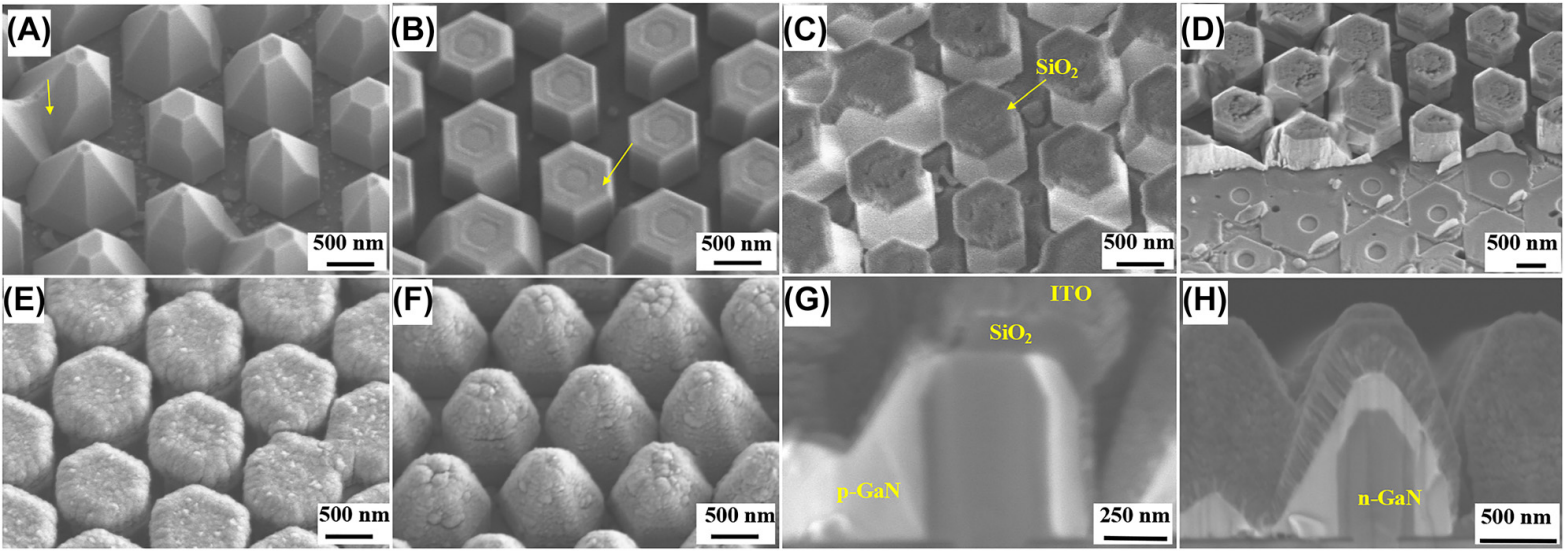
SEM characterizations of the NWs during fabrication process. Tilted-view SEM images for the (A) as-grown NW structures; (B) NWs with (0001) plane truncated by dry etching in samples d, e, and f; (C) NWs with SiO_2_ insulator formed on the apex for samples d, e, and f; (D) exposed n-electrode region by ICP dry etching of NWs; (E) ITO deposition on samples d, e, and f; (F) NWs of samples a, b, and c covered by ITO. Cross-sectional view SEM images of the NWs (G) with and (H) without (0001) plane etching, wherein the p-GaN shell, SiO_2_, ITO, and n-GaN core regions are marked.

According to the characterization results by SEM in our previous work [34], the (0001) plane MQS at the apex region usually manifests a serpentine shape, resulting in the formation of a few screws and Frank-type partial dislocations during p-GaN growth. Such defects in the p-GaN shell and the relative-low quality of (0001) plane MQS can be removed by etching off the apex region. To confirm the effect, NW-LEDs with different chip sizes were fabricated on the as-grown samples without and with removing the apex region. Figure 2(A) shows the tilted-view SEM images of the as-grown structures, and the pyramidal shape of the NWs is observed after p-GaN shell growth. Nevertheless, some NWs were connected due to the relatively high growth rate of p-GaN shell and sufficient precursors supply during MOVPE growth. For samples d, e, and f, the NWs were subjected to an ICP dry etching process to truncate the (0001) plane apex region, as confirmed in Figure 2(B). The formed hexagonal circles in the exposed area of each NW can be referred to as the cross-sectional MQS structures, indicating that the etching rates were different for the n-GaN core, MQS, and p-GaN shell under the identical conditions. Figure 2(C) shows the acquired SEM image of the NWs deposited with SiO_2_ insulating film on the truncated apex region, while the SiO_2_ on the sidewall was post-cleaned by dilute BHF solution. Here, it is worth mentioning that the thickness of SiO_2_ on the sidewall is much lesser than in the apex region due to the intrinsic feature of sputtering deposition. Subsequently, the NWs and SiO_2_ marks layer in the n-electrode area of all the samples were removed by dry etching to exposure the n-GaN substrate, as shown in Figure 2(D). Figure 2(E) and (F) depict the NWs covered with an ITO layer in the samples with (d−f) and without (a−c) apex (0001) plane etching, respectively. The cross-sectional morphology of NWs was also inspected to confirm the apex etching and deposition of ITO layer, as shown in Figure 2(G) and (H) From Figure 2(G), the bright contrast indicates the p-GaN, n-GaN core, and an ITO layer, while the (0001) plane region was etched and covered with SiO_2_ as designed. Finally, all the samples underwent a thermal annealing process of the ITO layer followed by the Cr/Ni/Au films deposition for n- and p-electrodes.

### 3.2 Effect of the NW apex region on the device properties

**Figure 3:**
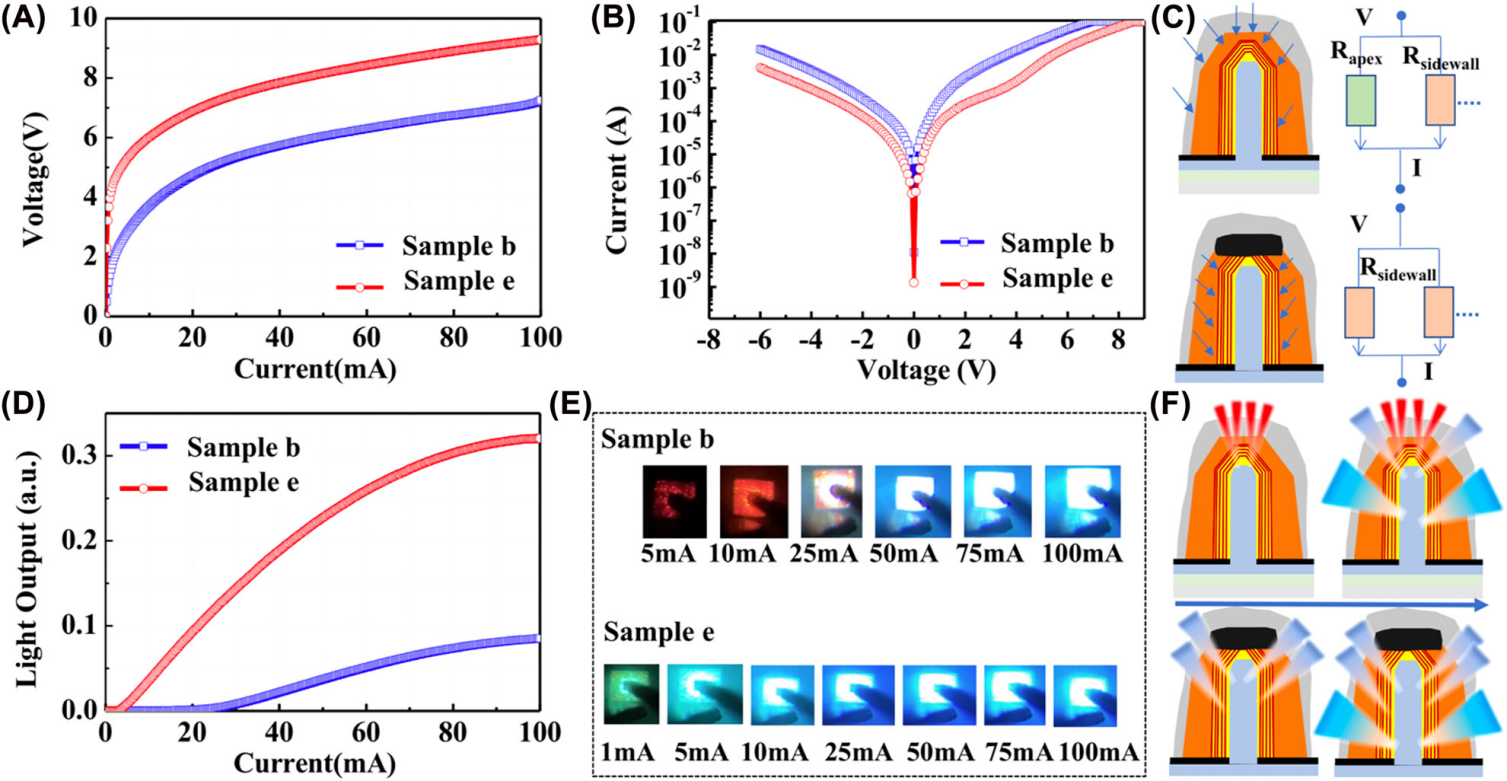
*I*–*V*–*L* curves, luminescence photos, and schematic images of the current injection paths in samples b and e. (A) *I*–*V* characteristic in samples b and e; (B) the corresponding *V*–*I* curves plotted in semi-log scale measured from −6 to 9 V; (C) schematic distribution of current injection and corresponding circuits in samples b and e under the same voltage. (D) The current-light output of samples b and e as a function of injection current; (E) the luminescence photos in samples b and e, captured at different injection currents; (F) schematic diagrams of the emission region in NWs with and without (0001) plane MQS, at low and high injection currents.

The effect of the (0001) plane MQS region on the device properties was assessed by comparing the electronic and optical features in samples b and e with a chip size of 100 × 100 μm^2^. Despite the etching and insulating of the NW apex region in sample e, the growth conditions and the other device fabrication processes were the same in both samples. Figure 3(A–F) show the *I*–*V* curves under forward current injection, the corresponding semi-log *V*–*I* curves measured from −6 to 9 V, the light output as a function of injection current, and the luminescence photographs, respectively. As shown in Figure 3(A), the threshold voltage was increased from 1.5 V for sample b to 3.9 V for sample e, and no significant difference was observed in the differential resistance. For sample b, under a low current range, it was dominantly injected into the (0001) plane MQS region, which involves a higher InN fraction and few screws or partial dislocations. Therefore, emission color variation and leakage path might exist, leading to the lower threshold voltage in sample b. To verify such speculation, the *V*–*I* characteristics under voltage-driven are plotted in semi-logarithm as a function of voltage, as presented in Figure 3(B). The reduced current leakage in sample e is evident as the current value decreases from 1.5 × 10^−2^ A to 4.0 × 10^−3^ A at a reverse bias of −6 V. It is demonstrated that the leakage point located at the (0001) plane region was slightly reduced by etching the (0001) plane region of the NWs. Nevertheless, the nonuniform p-GaN shape of the NWs and a few imperfect coverages by the SiO_2_ insulator on the (0001) plane apex still gave rise to the residual high leakage current, which is much higher than the current value of 1 × 10^−7^–10^−9^ A in the conventional planar LEDs at the same reverse bias region [42–45]. Therefore, a further suppression of the current leakage, such as protection of the etched surface in the (0001) plane region, is necessary. In addition, from the curves under forward bias in Figure 3(B), the calculated resistance is slightly higher in sample e. This is attributed to the etched surface on (0001) plane region and covered with SiO_2_ film, which caused different current injection paths along the NWs. In addition, the thickness of p-GaN shell spatially varied due to the slower growth rate on (1–101) plane than that of (0001) and (10–10) planes.

Figure 3(C) illustrates the current injection paths and corresponding equivalent circuits for samples b and e under the same applied voltage to elaborate the phenomenon. The lower resistance in the (0001) plane region results in the lower resistance of the whole NWs in sample b. A 3.8-fold enhancement of the light output was confirmed in sample e at an injection current of 100 mA, as shown in Figure 3(D). The light output curve features with a turning point around the injection current of 25 mA, which is attributable to the different emitting areas with an increase in injection current. To confirm the light emission, luminescence photographs were captured from the front side of the devices through a microscope. As shown in Figure 3(E), both samples can visually confirm luminescence even below 25 mA. However, below 25 mA, the emission in sample b was dominant in red color and appeared to be bright blue as the injection current increased. For sample e with (0001) plane etching, green–blue light emission was observed even at the low injection current of 1 mA. The variation of emission area from low to high injection currents is illustrated in Figure 3(F). As the current increases, it can reach the lower part of the NWs with low In content, resulting in light blue luminescence. The results suggest that red emission at a low injection current can be suppressed by etching off the (0001) plane apex region of the NWs. However, the reverse leakage remained high in the NW-LEDs with truncated (0001) plane MQS, which also induced the nonradiative recombination centers or nonuniform coverage by SiO_2_ dielectric film. The ICP dry etching commonly introduced surface damage, dangling bonds, and other types of surface defects on the (0001) plane region. Therefore, it is necessary to improve the performance of the devices by further optimizing the etching method to minimize the damage and applying surface passivation by atomic layer deposited Al_2_O_3_ dielectric films [46–48]. The introduction of an electron blocking layer or selective H_2_ plasma treatment to suppress the emission from the (0001)-plane of the as-grown NW might also be promising to improve the emission properties in NW-LEDs without etching off the apex region [49]. Moreover, the extinction of the (0001)-plane prior to the MQS growth is also preferred to reduce the emission at the tip region [50].

**Figure 4:**
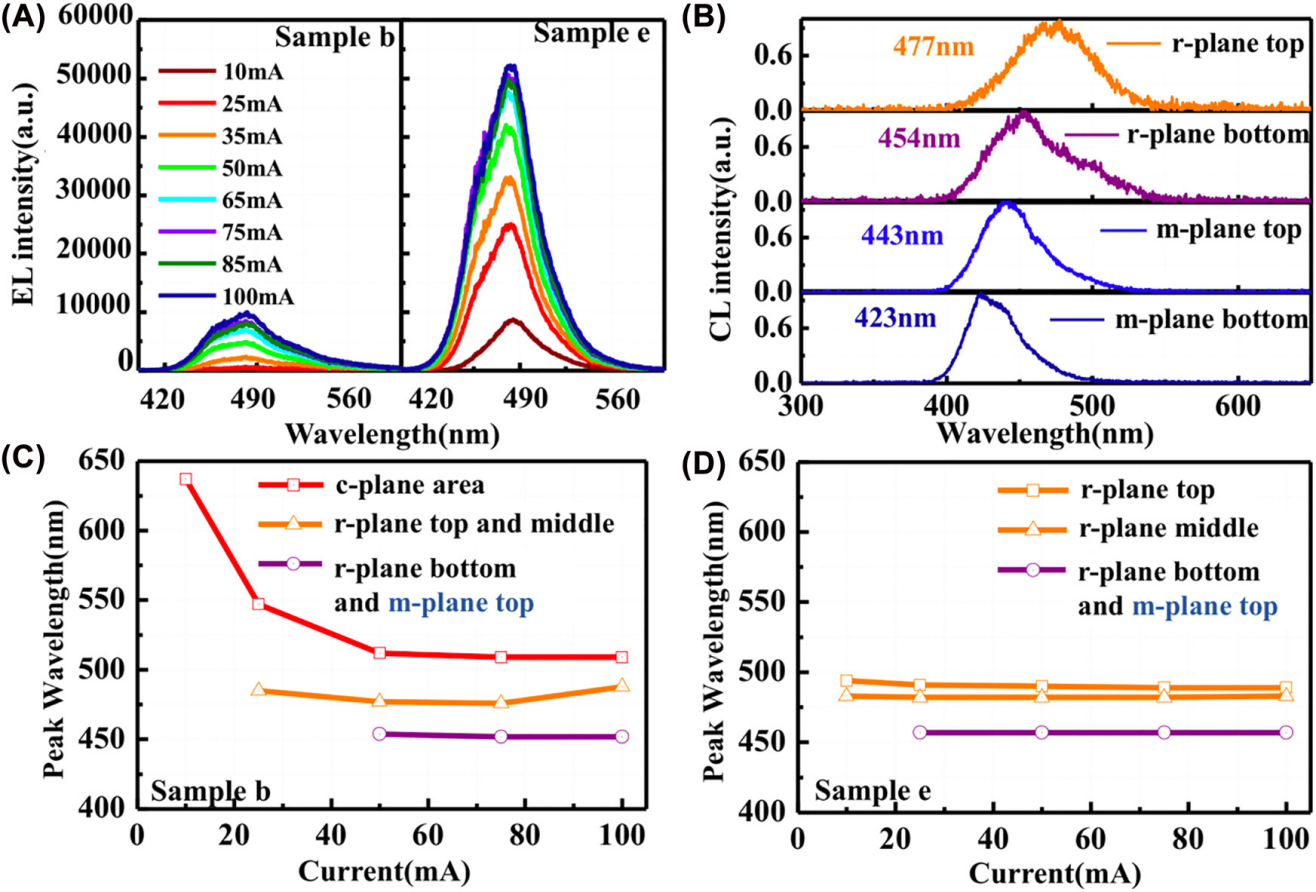
Comparison of EL spectra and the corresponding CL measurement results of samples b and e. (A) EL spectra in sample b and e measured under different injection current. (B) CL spectra acquired at (1–101) plane top, (1–101) plane bottom, (10–10) plane top, and *m*-plane bottom of the NWs. Panels (C) and (D) show the EL peak wavelengths derived from Gaussian fitting at each injection current for samples b and e, respectively. The emission peaks are assignable to different positions of the NWs. The (1–101) and (10–10) planes of NWs are indicated as “*r*-plane” and “*m*-plane” in the figures, respectively.

The corresponding EL spectra under different injection currents were acquired for samples b and e, as plotted in Figure 4(A). It is confirmed that the EL intensity is increased by a factor of 5.2 for sample e with (0001) plane etching. To correlate the EL emission peaks with different regions of the NWs, the CL spectra were measured at the top and bottom areas of (1–101) and (10–10) planes in one NW. As indicated in Figure 4(B), the emission wavelength was blueshift (∼20 nm) from the top to bottom in each plane of the NW, while the (1–101) plane region is featured with a longer emission wavelength (477–454 nm) than that of the (10–10) plane (443–423 nm). Here, the CL emission signal of the (0001) plane area was undetectable because the intensity was extremely low. The peak wavelength of the EL spectra is slightly longer than that of the CL results due to the differential features of CL and EL measurements. The CL measurement was carried out on individual planes of one NW, while EL was acquired from all planes of entire NWs in the mesa area. Multi-peak Gaussian fitting was applied to assign the EL emission to different positions of the NWs. Figure 4(C) and (D) show the fitted peak wavelengths as a function of injection current for samples b and e, respectively. Compared with the CL emission peaks, it can be deduced that the three EL peaks in sample b are assignable to the (0001) plane area, (1–101) plane top and middle area, and (1–101) plane bottom and (10–10) plane top area, respectively. Nevertheless, the EL emission of sample b was mainly originated from the apex region of NWs under the low injection current. In addition, the emission peak from the (0001) plane MQS region exhibits a blueshift from 638 to 525 nm with an increase in injection current, as shown in Figure 4(C). The three Gaussian peaks in sample e are associated with the (1–101) plane top, (1–101) plane middle, (1–101) plane bottom, and (10–10) plane top regions. The emission peaks are quite stable versus injection current, which is completely consistent with the intrinsic characteristic of semipolar and nonpolar planes. From the above results, with ICP etching of the (0001) plane MQS at the apex region of the NW, the reverse leakage and the weak emission of the (0001) plane region were suppressed, and the current could be sufficiently injected into the sidewall of the NWs.

### 3.3 Emission properties of NW-LEDs with different chip sizes

**Figure 5:**
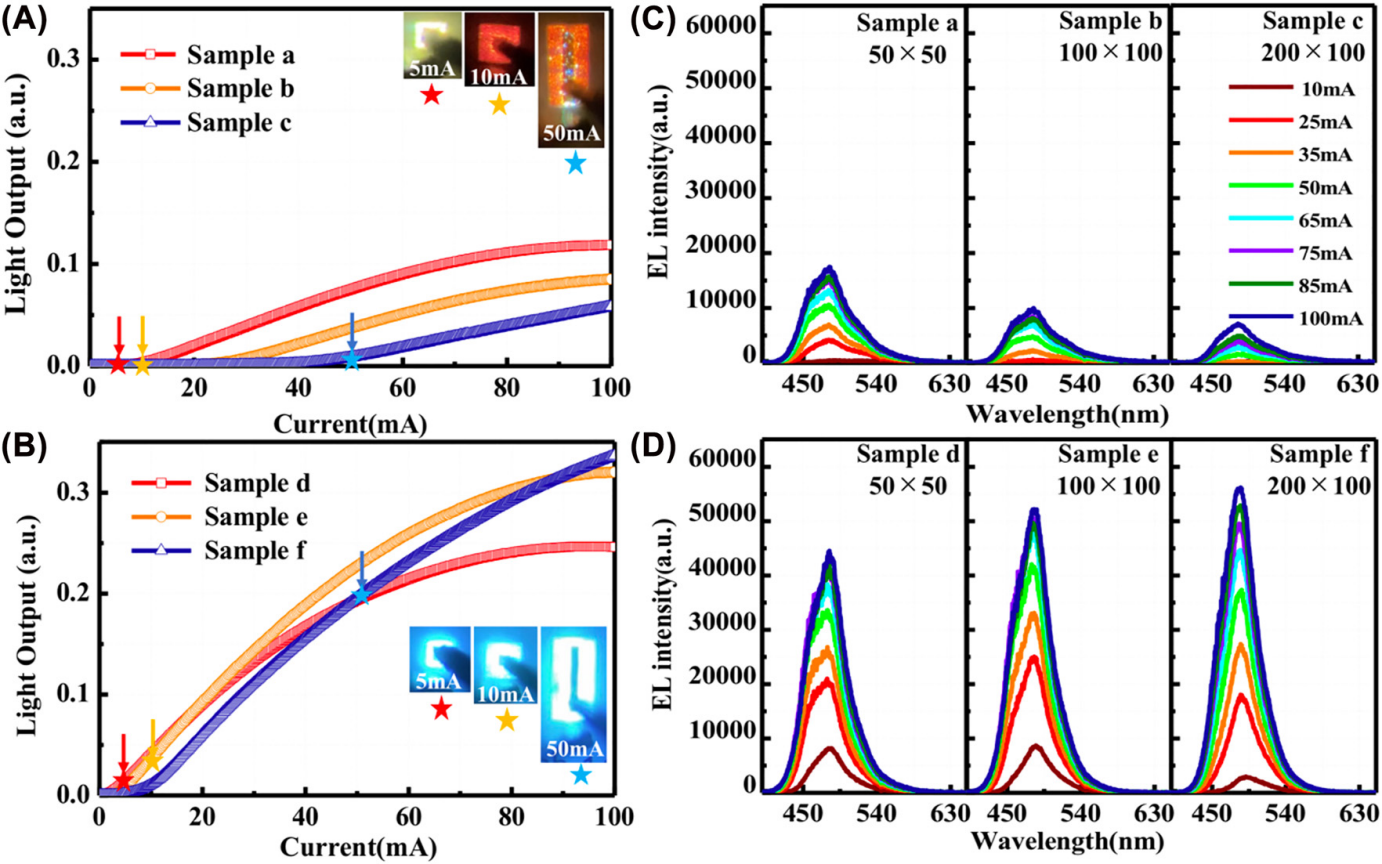
Light output and EL spectra of the samples with different chip sizes. (A) Light outputs of NW-LEDs in samples a, b, and c without (0001) plane etching. The insets show the optical microscopy emission photos taken at 5, 10, and 50 mA for samples a, b, and c, respectively. (B) Light outputs of samples d, e, and f with (0001) plane etching are plotted as a function of injection current. EL spectra of (C) samples a, b, and c without (0001) plane etching and (D) samples d, e, and f with (0001) plane dry etching at the NW apex region.

Two types of NW-LEDs with different mesa sizes of 50 × 50 µm^2^ (samples a and d), 100 × 100 µm^2^ (samples b and e), and 100 × 200 µm^2^ (samples *c* and f) were prepared, wherein the (0001) plane etching was performed in samples d, e, and f. Figure 5(A) and (B) present the light output of the two batches of samples without and with (0001) plane etching, respectively. The light outputs in samples d, e, and f were enhanced by removing the (0001) plane MQS region. Specifically, under low injection currents, the emission is rather weak for samples a, b, and c. Such phenomenon is more prominent for sample c below the injection current of 50 mA. With a larger mesa area, an increased current was required for the localization at the apex region of each NW. As shown in the insets of Figure 5(A) and (B) the samples a, b, and c exhibited reddish emission under injection current of 5, 10, and 50 mA, respectively, while the emission colors of samples d, e, and f were dominant by light cyan. Therefore, the effect of (0001) plane MQS on the emission properties of NW-LEDs is further confirmed. Nevertheless, the thermal saturation was observed in the samples with (0001) plane etching as the chip size reduced. This is attributed to the high current density and high resistance of the thick p-GaN on the (10–10) plane area. The EL spectra were simultaneously measured for all the samples under different injection currents. The main emission peak beyond 20 mA is located at around 485 nm for all the samples. The high effective current density for the samples without etching gave rise to the stronger EL emission as the mesa area decreased, which is in regardless of the sidewall effect that commonly observed in planar micro-LED [51]. Under the same current injection, the current density increases with a decrease of the mesa area. As a result, the current can be injected to the lower part of the NWs, wherein the (1–101) and (10–10) planes generally have higher crystalline quality and luminous efficiency. A contrary tendency was observed in samples d, e, and f, as shown in Figure 5(D). Overall, the EL intensity of the samples with truncated (0001) plane region was enhanced up to 7.9 times. The EL intensity increased as the mesa area increased in samples d, e, and f, specifically under the injection current beyond 30 mA. This is associated with the suppressed current injection into the (0001) planes, which promotes the light emission from (1–101) and (10–10) planes, resulting in the increased intensity as the number of NWs increases. In addition, as aforementioned, the thermal saturation also dispatched a part of the injection current in the samples with a smaller chip size. Therefore, it indicates that the ICP etching-induced nonradiative recombination centers need to be reduced to further increase the performance of the NW-LEDs.

### 3.4 Discussion of the electronic-optical characteristics

**Figure 6:**
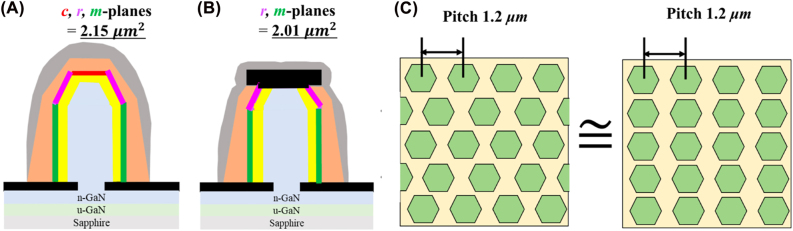
Definitions for the equivalence luminescence area of NWs. (A) Luminescence area (2.15 µm^2^) per NW in samples a, b, and c [here, (0001), (1–101), and (10–10) planes of NWs are indicated as “*c-, r*-, and *m-*plane”]; (B) luminescence area (2.01 µm^2^) per NW in samples d, e, and f with truncated apex; (C) approximation method for the number of NWs.

As the luminescence area in samples a, b, and c differs from that with dry etching off the (0001) plane region, the effective emission region and current density were considered to elaborate the electronic-optical characteristics. In this case, the current-voltage optical characteristics in terms of current density were derived from the equivalent luminescence area of the entire NW surface. Figure 6(A) and (B) show the schematic illustrations used to define the effective luminescence area of NWs without and with the truncated apex. The total area of one NW in samples a, b, and c was calculated to be 2.15 µm^2^ by summing up all the emission facets, including one hexagonal (0001) plane, six trapezoidal (1–101) planes, and six rectangular (10–10) planes. Regarding the case for samples d, e, and f without the (0001) plane, the effective emission area is slightly reduced to 2.01 µm^2^. To simplify the calculation of overall NWs in the devices, the triangular arranged NW arrays were approximated to square alignment with same pitch of 1.2 µm, as illustrated in Figure 6(C). Based on this approximation, the number of NWs in p-electrode and mesa areas can be easily derived for all the samples. The number of NWs in each ITO area was derived by multiplying the vertical and horizontal values. Therefore, the luminescence area of samples a, b, c, d, e, and f are calculated to be 3740, 15000, 29900, 3490, 14000, and 27900 µm^2^, respectively, via multiplying the emission area per NW by the number of NWs.

**Figure 7:**
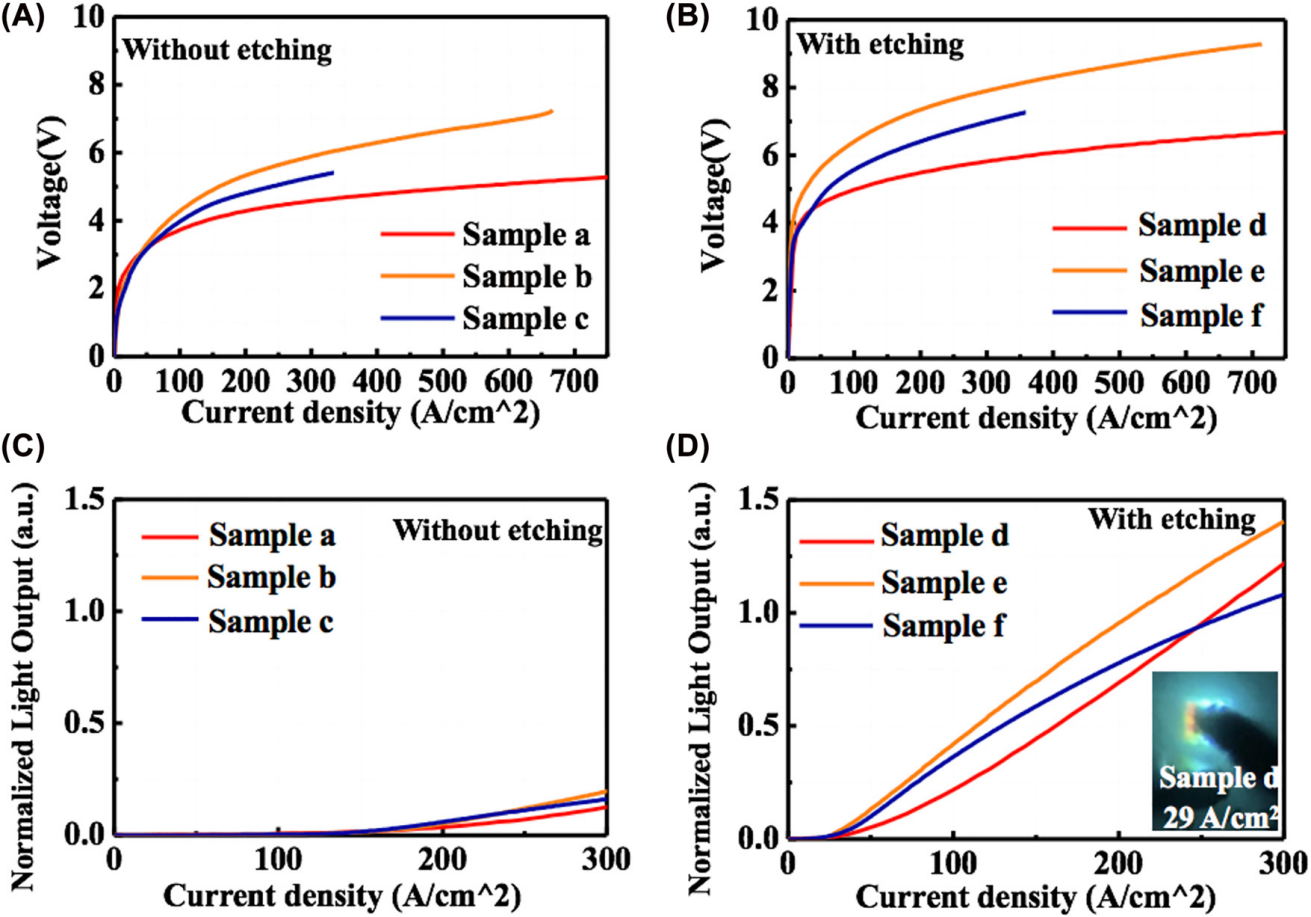
*I*–*V* characteristics in the samples (A) without and (B) with etching as a function of effective current density. Normalized light outputs of (C) samples (a, b, and c) without etching and (D) truncated samples (a, b, and c), while the range of current density is set to 0–300 A/cm^2^. The inset photo shows the emission of sample d under the current injection of 29 A/cm^2^.

To precisely analyze the results, the *I*–*V* curves were re-plotted as a function of current density, which was determined using the effective emission areas of overall NWs, as presented in Figure 7(A) and (B). A similar variation trend of the operation voltage is observed in the NW-LEDs regardless of whether the (0001) plane region was etched or not. This is attributable to the different p-electrode areas, resulting in the different ratios of p-electrode to mesa in the samples (0.64, 0.16, and 0.27), as described in Table 1. The higher coverage of p-electrode in the mesa area can promote the current spreading and reduce the operation voltage. Comparing the samples with and without (0001) plane region etching, the observed enhancement of threshold voltage was caused by the suppression of initial current flowing into the (0001) plane MQS region. Regarding the case of the light output, the value was normalized by dividing their effective chip size. Figure 7(C) and (D) show the normalized light output as a function of the effective current density, which are plotted from 0 to 300 A/cm^2^. It can be observed that the emission intensity of the truncated samples was enhanced by more than ten times under the same current density as compared to samples a, b, and c. Specifically, different from the trends as observed in Figure 5(C), the normalized emission intensity of sample e appears to be higher than that of the samples d and f. This is ascribed to the lower ratio of p-electrode to mesa in sample e, which enables higher collection of emission light during measurements. As shown in the luminescence photograph inset in Figure 7(D), the orange color is slightly observed in sample d with the current injection density of 29 A/cm^2^, indicating that a few (0001) plane regions with low efficiency remain. A peak internal quantum efficiency (IQE_peak_) of 25% at the low injection current of 18 mA was estimated for sample e from the current-light output curve, which were fitted with the carrier rate equations [*ABC* – *f*(*n*) model]. Here, the *f*(*n*) represents the recombination process outside the active layer, and more detailed information can be found in the paper published by Han et al. [10]. Specifically, in this model, the IQE_peak_ is given by *B*/(*B* + 2(*AC*)^0.5^) regardless of *f*(*n*), and the IQE_peak_ can be obtained by fitting the current-light output curve in the current range of *I* < *I* at IQE_peak_. For the samples without etching, the light output was rather low and required larger current than the etched ones to reach the identical emission intensity, which made it difficult to calculate the IQE_peak_ with such fitting model. In addition, the complex recombination mechanisms in NW-LEDs depending on the injected current density with varied emission region along the NWs and leakage current are intricately tangled in the samples without etching. Nevertheless, it is considered that uniform and precise etching of the apex region is preferred to improve the emission efficiency in NW-LEDs. Further optimization of the electrode configuration is also expected to increase the emission intensity in NW micro-LEDs.

## 4 Conclusions

To improve the emission efficiency of GaInN/GaN MQS NW-LEDs, we fabricated devices by ICP dry etching off the (0001) plane MQS region, which featured with low crystalline quality and luminescence intensity. Dry etching the *c*-plane region with ICP allowed suppression the orange–red emission from the (0001) plane MQS region. As a result, even at a low injection current of 5 mA, the dominant emission by the (1–101) and (10–10) planes MQS was achieved, while the EL intensity was enhanced up to 7.9 times. Furthermore, the current leakage was slightly improved by removing the (0001) plane region regardless of the ICP etching-induced surface damage. Same phenomenon was observed for the NW-LEDs with different chip sizes, i.e., the EL intensity decreased as the mesa area increased when under the injection current below 30 mA. Specifically, with the same injection current density, the emission intensity of the truncated samples was enhanced for more than ten times as compared to the reference samples. The observed enhancement of threshold voltage in the samples with (0001) plane etching was caused by the suppression of initial current injected into the (0001) plane MQS region. Moreover, it was found that a higher coverage of p-electrode in the mesa area can promote the current spreading and reduce the operating voltage. The results indicated that the (0001) plane etching method effectively suppress the weak emission in the apex region of GaInN/GaN MQS NWs and is promising for realizing high-efficiency NW-based micro-LEDs with high emission stability. Nevertheless, precise etching and passivation of the apex region are necessary to further reduce the reverse leakage current and improve the emission efficiency in NW-LEDs.
